# *Leptospira interrogans* Serogroup Pomona in a Dairy Cattle Farm in a Multi-Host Zootechnical System

**DOI:** 10.3390/vetsci9020083

**Published:** 2022-02-16

**Authors:** Antonino Aliberti, Valeria Blanda, Vincenzo Di Marco Lo Presti, Giusi Macaluso, Paola Galluzzo, Cristina Bertasio, Carmela Sciacca, Francesca Arcuri, Rosalia D’Agostino, Dorotea Ippolito, Flavia Pruiti Ciarello, Alessandra Torina, Francesca Grippi

**Affiliations:** 1 Istituto Zooprofilattico Sperimentale della Sicilia, 90129 Palermo, Italy; antoninoaliberti.vet@gmail.com (A.A.); vincenzo.dimarco@izssicilia.it (V.D.M.L.P.); paola.galluzzo@izssicilia.it (P.G.); sciacca.carmela@gmail.com (C.S.); francescaarcuri11@gmail.com (F.A.); rosalia.dagostino1@gmail.com (R.D.); ippolito.dorotea@libero.it (D.I.); pruitiflavia@outlook.it (F.P.C.); alessandra.torina@izssicilia.it (A.T.); francesca.grippi@izssicilia.it (F.G.); 2 Istituto Zooprofilattico Sperimentale della Lombardia e dell’Emilia-Romagna, 25124 Brescia, Italy; cristina.bertasio@izsler.it

**Keywords:** *Leptospira interrogans* serogroup Pomona, cattle, outbreak, Sicily

## Abstract

Bovine leptospirosis is an infectious zoonotic disease causing reproductive problems and economic losses in livestock. This work reports, for the first time in Sicily (South Italy), an outbreak of *Leptospira interrogans* serogroup Pomona that occurred in cattle farms within the Nebrodi Park and was mainly characterized by full-term abortion. Blood and urine samples were collected at different time points from animals of six different farms (Farms A–F) sharing the same grazing area. Research of antibodies against pathogenic *Leptospira* species in serum samples was carried out via Micro Agglutination Test (MAT). Urine samples were subjected to pathogen isolation and molecular analyses via TaqMan Real Time-PCR. Genotyping of *Leptospira* species was obtained by Multi-locus sequence typing. MAT detected antibodies against *Leptospira interrogans* serogroup Pomona in serum samples of all the farms. Pathogenic *Leptospira* spp. DNA and culture isolation was obtained from urine samples. Genotyping confirmed the excretion of *L. interrogans* serogroup Pomona. This study describes clinical manifestations, diagnostic implications and epidemiological characteristics of an outbreak in cattle due to *L. interrogans* Pomona in a protected multi-host area, where domestic and wild animals share the same habitat, suggesting a role of wild species in transmission and persistence of Pomona serogroup among cattle.

## 1. Introduction

Leptospirosis is a zoonosis with a worldwide distribution [1,2]. It is caused by pathogenic helical spirochetes of the *Leptospira* genus (family Leptospiraceae, order Spirochaetales). The pathogen may affect several species of domestic and wild animals as well as humans [3,4,5,6,7]. In susceptible hosts, the clinical manifestations range from severe conditions to mild febrile symptoms or asymptomatic conditions [8,9]. After the bacteremia, the pathogen is able to colonize the kidneys and it is released in the urine, which thus represent the most common contamination route for *Leptospira* species [10].

The epidemiology of leptospirosis is related to the presence of susceptible hosts, both maintenance and incidental [11]. Maintenance hosts generally do not develop clinic forms of the disease, but act as natural pathogen sources, highly influencing *Leptospira* spp. epidemiology [12]. Serogroups Icterohaemorrhagiae and Ballum are mainly associated with rodents [13,14,15,16], serogroups Pomona and Tarassovi with pigs and wild boars [11,16,17,18,19,20], Bratislava serogroup with horses [21,22] and Sejroe serogroup with cattle and sheep [12,23]. In recent years, some serogroups have emerged among wild and domestic animals, suggesting that changes in leptospirosis epidemiology may occur over time [24].

Examples of incidental hosts include companion animals (e.g., dogs and horses) [25] as well as livestock like cattle, pigs and horses. Dogs have been known to be hosts for pathogenic leptospires for over 80 years [26]. While infection was most commonly associated with the presence of antibodies to the serogroups Canicola and Icterohaemorrhagiae, it is now clear that dogs are susceptible to infection with a wide range of serovars. Based on the available antibody prevalence data, the major serogroups to which dogs in Europe seroconvert are Icterohaemorrhagiae, Grippotyphosa, Australis, Sejroe and Canicola [27]. Seroconversion of dogs to the serogroup Grippotyphosa is common in continental Europe, but appears to be rare in the UK and Ireland. This might be explained by the distribution of relevant reservoir hosts [27].

Bovine leptospirosis may be caused by a wide variety of serovars and represents a critical occupational zoonotic disease [28,29]. Cattle are maintenance hosts of Sejroe serogroup and, in particular, of Hardjo serovars. These consist of two serologically indistinguishable but genetically distinct strains: *Leptospira borgpetersenii* serovar Hardjo (Hardjobovis), the common strain of this serovar maintained in cattle, and *Leptospira interrogans* serovar Hardjo (Hardjoprajitno), widespread in some parts of the world. Both strains are able to colonize and persist in the genital tract of infected animals, suggesting that venereal transmission can also occur [30,31]. The urinary acid pH of cattle, together with the use of artificial insemination, may reduce the direct transmission of leptospirosis [32]. Incidental infection in cattle is caused by several serotypes belonging to the serogroups Icterohaemorrhagiae, Canicola, Hebdomadis, Sejroe, Pyrogenes, Autumnalis, Australis, Javanica, Tarassovi, Grippotyphosa and Pomona, with rare clinical symptoms, mainly represented by pyrexia, haemolytic anaemia, hemoglobinuria, jaundice and, occasionally, meningitis and death. Late abortion, stillbirth, premature birth or birth of weak and low-weight calves may also occur in the chronic form of leptospirosis. Infection with incidental serotypes in adult cattle often results in high abortion rates in the infected herd, occurring a few weeks after the acute phase of the disease. Recently, congenital jaundice in aborted fetuses has been included among the clinical signs of leptospiral abortion caused by incidental serotypes [33].

The Pomona serogroup is the second most widespread serotype in cattle in Italy [24], even if severe infections caused by Pomona serogroup in cattle are rare and mainly affect young animals [30]. Its prevalence is increasing in North-Central Italy [34] where the prevalent type of extensive farming has favored its spread due to contact with wild animals, in particular wild boars.

The present work aims to report, for the first time in Sicily, an outbreak of Leptospirosis due to *Leptospira interrogans* serogroup Pomona in a dairy cattle farm, located in a protected natural area of Sicily (Nebrodi Park). In this area, a complex multi-host ecosystem exists with a zootechnical system mainly characterized by mixed farms (cattle, sheep, goats, pigs, horses and donkeys, etc.) with extensive and transhumant breeding. These farms exploit municipal pastures, in which different domestic animal species from different farms share watering and feeding points with the rich local wild fauna (wild pigs, wild boars, foxes, martens, etc.). In addition, an uncontrolled increase of wild and/or feral pigs has been reported in the last decades. Sharing of habitats increases contacts among species, enhancing the risk of infectious disease interspecies transmission. In such a complex multi-host ecosystem, an outbreak of Leptospirosis due to *Leptospira interrogans* serogroup Pomona in an accidental host (cattle) is reported for the first time, and the epidemiological, clinical and diagnostic aspects are discussed.

## 2. Materials and Methods

### 2.1. Farms

**Figure 1:**
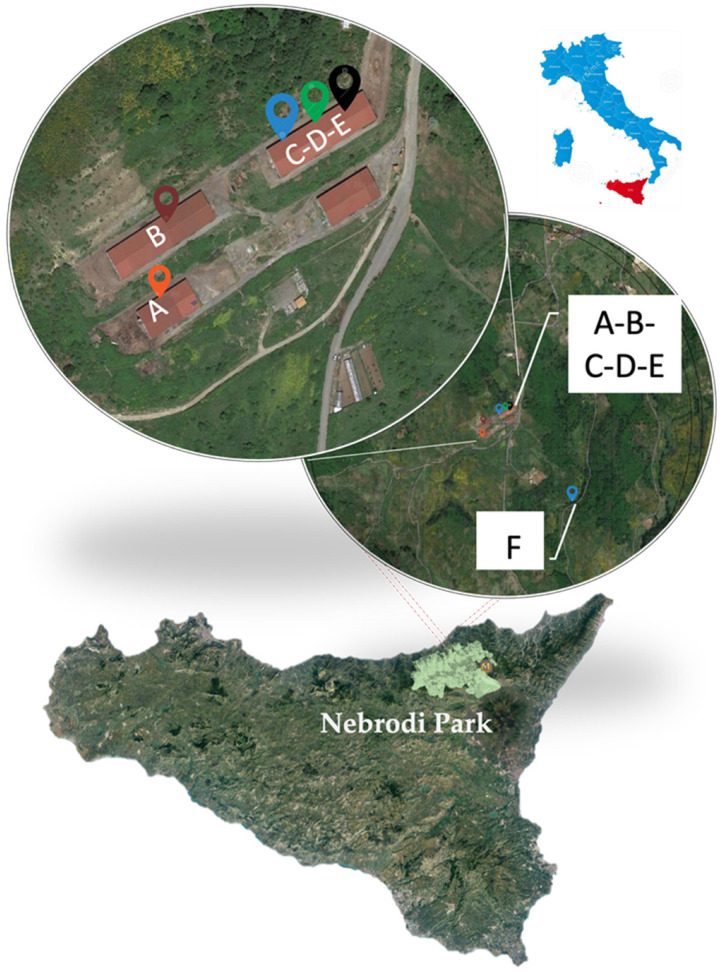
Farms localization within the Nebrodi Park (Sicily, Italy).

Outbreak identification occurred in January 2019 in a dairy herd (Farm A) in the Nebrodi Park, a protected area located in Northeastern Sicily (South Italy), following the report of abortions and fertility disorders. The farm was included in a livestock production facility consisting of four municipal housing structures shared among different breeders, located at a distance of 500 m from each other (Figure 1).

Farm A consisted of 33 Simmental cattle and extended over an area of about 1000 square meters, separated from the other structures by a difference in height and walls. The farm had an independent water supply and a biosecurity plan. Lactating animals were sheltered at night and led to pasture during the day in a surrounding 3 km radius area. Contacts with other species, such as dogs, domestic pigs and wild boars, the latter endemic in the area, were likely.

The other farms, B (52 heads), C (17 heads), D (2 heads) and E (2 heads), sited in separate sheds of the same zootechnical complex, were considered as the same epidemiological unit. All farms had a semi-wild farming system of cow/calf for meat production, grazed in promiscuity with other animal species and sheltered in the coldest periods of the year. The unit did not present biocontainment plans. The grazing land and the water sources fall within the same perimeter as farm A, but watering points were not shared, although the presence of small ponds in the field, where all animals could drink from, or water streams shared between cattle and wildlife were transiently present, especially during the winter season, when the rains were more frequent. The last herd, Farm F (66 heads), was located about 1.2 km from the livestock complex, but it was included in the same epidemiological unit since its grazing areas were contiguous with herd A. The herd contained a nucleus of Angus breed cattle from Eastern Europe introduced about 2 years before.

No vaccination protocols for leptospirosis were adopted in the farms.

### 2.2. Sampling

Blood samples were obtained by venipuncture of the coccygeal vein. In farm A, 33 cows were sampled at three different time points: on day 0 (T0), which occurred seven days after the first signs of abortion were noticed, on day 90 (T1), which occurred after antibiotic treatment, and on day 120 (T2). In farm B, the 52 cattle were sampled at T0 and T1 without having received any antibiotic treatment, while in farms C, D, E and F a unique blood sampling was carried out on all the animals (T0).

Urine samples were collected by spontaneous urination to perform *Leptospira* species detection by culture and molecular methods. In detail, for farm A, 16 and 24 urine samples were collected at T0 and T1, respectively; for farm B, 2 and 20 urine samples were collected at T0 and T1; in farms C, D and E, respectively, 3, 2 and 2 urine samples were taken, while no urine samples were collected in farm F. No urine samples were collected at T2.

Milk samples were collected from all the cows of farm A and were subjected to *Leptospira* spp. research by culture examination and molecular methods. In addition, two aborted fetuses (heart, lung, brain, spleen, liver), together with the placenta and utero-vaginal discharge, were collected from herd A and subjected to culture examination and DNA research of *Leptospira* species.

Soil and water samples and the blood of a dog present in farm A were also sampled and subjected to molecular analyses.

### 2.3. Differential Diagnosis

In order to determine the cause of abortion, different serological tests against the main abortion agents were performed.

The serological response to *Brucella* spp. was assessed by Rose Bengal Test (RBT), and Complement Fixation Test (CFT), according to standard OIE procedures [35].

All serum samples were tested for antibodies against *Coxiella burnetii*, *Neopsora caninum*, Infectious Bovine Rhinotracheitis or bovine herpesvirus (IBR or BHV) and Bovine Viral Diarrhoea (BVD) by enzyme-linked immunosorbent assays (ELISA) using commercial test kits and following the manufacturer’s instructions. The corresponding values for optical density were recorded by a 96-well microplates reader. Specific details of the ELISA kits, along with the sensitivities and specificities of the assays, are shown in Table 1.

### 2.4. Serological Test for Leptospirosis

The gold standard microscopic agglutination test (MAT) was carried out on all the serum samples according to the OIE Manual of Terrestrial Animals [2,36].

Cultured *Leptospira* spp. strains belonging to the eight pathogenic serogroups circulating in Italy were provided by the National Center for Leptospirosis (IZS LER, Brescia, Italy) and were used for sample agglutination [16]. In particular, they included *L. interrogans* serogroup Australis serovar Bratislava, *L. interrogans* serogroup Pomona serovar Pomona, *L. kirschneri* serogroup Grippotyphosa serovar Grippotyphosa, *L. borgpetersenii* serogroup Ballum serovar Ballum, *L. interrogans* serogroup Sejroe serovar Hardjo, *L. borgpetersenii* serogroup Tarassovi serovar Tarassovi, *L. interrogans* serogroup Icterohaemorragiae serovar Copenhageni and *L. interrogans* serogroup Canicola serovar Canicola.

*Leptospira* spp. cultures were performed in Ellinghausen–McCullogh modified by Johnson–Harris (EMJH) culture medium, obtained combining the *Leptospira* Enrichment EMJH reagent and the *Leptospira* Medium Base (Becton Dickinson spa, Italy).

A MAT cut-off of 1:100 was used to identify positive samples and two-fold serial dilutions starting from 1:100 up to 1:6400 were used for titration of positive sera. The titer showing at least 50% agglutination of leptospires was considered the sample titer.

### 2.5. Culture Examination/Isolation of Leptospira *spp.*

The isolation procedure was performed according to the OIE [36], using selective liquid EMJH and selective semisolid EMJH. Each urine sample was collected in EMJH medium (dilution 1:10) selective for *Leptospira* species and delivered to the Laboratory at room temperature within six hours from collection. Each urine inoculum was further diluted with selective liquid EMJH (dilution 10^−2^) and with selective semisolid EMJH (dilutions 10^−3^ and 10^−4^). Abortion tissue samples were transported at room temperature, the outer part was flamed and 1 g of sample was withdrawn, and 9 mL of sterile physiologic solution was added and manually homogenized. Subsequently, 1 mL of homogenate (dilution of 10^−1^) was subjected to further dilutions (up to 10^−4^) as described for urine samples.

Inocula were checked every 20 days under a darkfield microscope with a 10 × objective. To define a negative sample, inocula were further renewed in liquid and semi-solid selective EMJH, up to a maximum of six months. *Leptospira* positive isolations were subjected to molecular investigation.

### 2.6. Molecular Tests for Leptospirosis

Molecular investigations were carried out on urine samples, urine inocula, placenta, utero-vaginal discharge, milk, soil and water samples. For DNA extraction from placenta, the surface was flamed and 1 g of tissue withdrawn and homogenized in 9 mL of sterile physiological solution with Stomacher^®^ 80 Biomaster (Seward Limited, London, UK).

Lysozyme 10 mg/mL (Roche, Linscott, USA) was added to each sample and incubated at 37 °C for 30 min. DNA extraction was carried out using the Purelink Genomic DNA Kit (ThermoFisher Scientific, Rodano, Italy) according to the manufacturer’s instructions. For each sample, the extraction internal control (IC) included in the Quantifast Pathogen + IC Kit (Qiagen, Hilden, Germany) was used. A Taqman-based Real Time-PCR was carried out to detect pathogenic *Leptospira* species by amplifying a *lipL32* gene fragment, which encodes the outer membrane protein Lipoprotein L32, present only in pathogenic *Leptospira* species [37].

Primers and probe sequences are reported in Table 2. Sterile physiological solution was used as a negative extraction control. Sterile MilliQ water and DNA extracted from one of the eight pathogenic *Leptospira* cultured strains were used as negative and positive amplification control, respectively.

The amplification program included a denaturation step (95° C for 5 min) and 45 amplification cycles (denaturation at 95 °C for 15 s and annealing and extension at 60 °C for 30 s).

### 2.7. MLST and Phylogenetic Analyses

Genotyping of *Leptospira* species was carried out by the Multi-locus sequence typing at the Italian National Reference Centre for Leptospirosis at the Istituto Zooprofilattico Sperimentale della Lombardia e dell’Emilia Romagna, Brescia (Italy) [38,39,40,41]. To genotype leptospires, seven housekeeping genes, UDP-N-acetylglucosamine pyrophosphorylase (*glmU*), UDP-N-acetylglucosamine pyrophosphorylase (*pntA*), 2-oxoglutarate dehydrogenase E1 component (*sucA*), triosephosphate isomerase (*tpiA*), 1-phosphofructokinase (*pfkB*), rod shape-determining protein rodA (*mreA*) and acyl-CoA transferase/carnitine dehydratase (*caiB*) were analyzed [39]. Assembled sequences were trimmed and aligned to allele reference sequences downloaded from the Bacterial Isolate Genome Sequence Database (BIGSdb) (available online: https://pubmlst.org/Leptospira/, accessed on 15 December 2021) to assign allele numbers to all seven loci. For strain identification, allelic profiles were queried against the *Leptospira* BIGSdb.

A phylogenetic tree was built using the concatemer of the seven MLST genes linked in the followed order: *glmU-pntA-sucA-tpiA-pfkB-mreA-caiB*. The evolutionary history was inferred using the Neighbor-Joining method [42]. The optimal tree with the sum of branch length = 0.18381177 is shown. The percentage of replicate trees in which the associated taxa clustered together in the bootstrap test (1000 replicates) are shown next to the branches [43]. The tree is drawn to scale, with branch lengths in the same units as those of the evolutionary distances used to infer the phylogenetic tree. The evolutionary distances were computed using the Maximum Composite Likelihood method [44] and are in the units of the number of base substitutions per site. All ambiguous positions were removed for each sequence pair (pairwise deletion option). Evolutionary analyses were conducted in MEGA X [45].

## 3. Results

### 3.1. Differential Diagnosis

Two animals from farm A resulted positive for *N. canimun* and one for *C. burnetii*. None of these showed signs of abortion.

Six animals showed a positive reaction for IBR specific gB and a negative reaction for IBR specific gE.

All the animals resulted negative for *Brucella* spp. and BVD.

### 3.2. Serological Results for Leptospirosis

In farm A, nine cows showed full-term abortion, followed by placental retention and reduced milk production. Out of the 33 animals, 11 (33.3%) resulted positive at MAT at T0, with 5 samples simultaneously positive for different *Leptospira* serogroups (*L. interrogans* serogroup Australis serovar Bratislava, *L. interrogans* serogroup Pomona serovar Pomona, *L. interrogans* serogroup Icterohaemorragiae serovar Copenhageni), with titers ranging from 1:100 to 1:400. At the second sampling (T1), subsequent to the antibiotic treatment of positive animals, 25 animals (75.8%) showed antibodies against *Leptospira* species, particularly against *L. interrogans* serogroup Pomona serovar Pomona. Antibody titers varied between 1:100 and 1:6400, with 15 animals with a titer ≥ 1:400. In detail, 9 treated animals showed an increased titer, while 15 of the 22 animals negative at T0 resulted positive. At T2, overall, 22 animals (66.7%) were serologically positive for *L. interrogans* serogroup Pomona, with 10 cows showing a reduction in the antibody titer and 5 becoming negative; only one cow negative at the previous sampling resulted positive (Table 3).

In herd B, serological investigation carried out at T0 detected 5 cows with antibodies against *Leptospira* species (Table 4), showing different serogroups (*L. interrogans* serogroup Australis serovar Bratislava, *L. interrogans* serogroup Icterohaemorragiae serovar Copenhageni, *L. interrogans* serogroup Sejroe serovar Hardjo and *L. interrogans* serogroup Pomona serovariant Pomona). Antibody titers ranged between 1:100 and 1:3200. At T1, 6 samples resulted positive, including 2 animals negative at the first sampling. All the serogroups belonged to *L. interrogans* serogroup Pomona, and titers ranged from 1:200 to 1:6400. Only 1 bovine, positive for *L. interrogans* serogroup Sejroe at the first screening, resulted negative at T1.

In Table 4, results of MAT are summarized for farm B obtained in the T0 and T1 sampling.

In Table 5, positive results at MAT are summarized for the farms C, D, E and F obtained in the only sampling, T0.

### 3.3. Leptospiral Molecular Detection and Isolation

**Figure 2:**
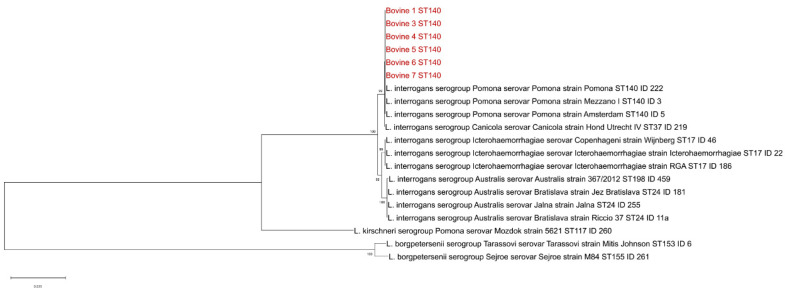
Phylogenetic tree based on concatenated sequences of the seven genes of the multi-locus sequence typing scheme. The DNA of urine sample with full MLST profile is indicated with its progressive number, the isolation year and its unique ID. The names of reference strains include the *Leptospira* species serovar and strain.

For farm A, the molecular investigation carried out on urine samples collected from serologically positive cows detected pathogenic *Leptospira* spp. DNA in all 11 samples (100%) analyzed at T0, and in 6 out of 24 samples (25%) at T1. Pathogenic *Leptospira* species were isolated from 5 positive urine samples (two from T0 and three from T1). Genotyping carried out in seven positive urine samples collected at T0 confirmed the excretion of *L. interrogans* serogroup Pomona. Phylogenetic analysis carried out using the concatemer of the seven MLST genes showed that Sicilian Leptospiral DNA samples clustered with the Pomona serovar (serogroup Pomona) reference strain (Figure 2).

Neither culture examination nor Real Time-PCR showed evidence of *Leptospira* spp. presence in samples of milk, water, soil, utero-vaginal discharge, placenta, aborted fetuses or from the dog serum.

For herd B, at time T0, one urine sample was positive at the isolation of *Leptospira* species; at T1, renal excretion of pathogenic *Leptospira* spp. DNA was detected in 5 out of 20 urine samples.

For breeding unit C, one urine sample out of the three collected ones allowed *Leptospira* species isolation. In the other farms, no detection by Real Time-PCR nor isolation of *Leptospira* spp. were showed.

In Table 6, results of Real Time-PCR, MLST and isolation in the acute phase are summarized.

## 4. Discussion

Numerous bacterial, viral, protozoan and fungal pathogens have been associated with infertility and abortion in cattle.

Determining the cause of abortion in cattle is difficult and a major challenge to the herd owner and veterinarian. Infectious agents represent the leading etiology, and the majority of diagnosed abortions are attributed to infections with the bacteria *Brucella abortus*, endemic in Sicily, and *Leptospira interrogans*, the protozoa *Neospora caninum* and the two viruses IBR and BVD [46]. Moreover, *Coxiella burnetii*, the causal agent of Q fever, which is a zoonotic disease, has been related to stillbirth, aborted fetuses and the delivery of weak and nonviable neonates in ruminants. Yet, the correlation between *Coxiella* seropositivity and abortion risk in bovines is far less understood [47,48].

These pathogens can result in substantial economic losses, indicating the need for control measures to prevent infection or disease.

This study describes an outbreak in cattle in Sicily, mainly characterized by full-term abortions. Although, from the differential diagnosis, two animals resulted positive for *N. canimun* and one for *C. burnetii*, none of them showed signs of abortion. Moreover, the animals with a positive reaction for only specific IBR gB indicated they were vaccinated with gE-deleted marker vaccines and not infected. All the animals resulted negative for *Brucella* spp. and BVD. Numerous animals tested positive to leptospiral diagnosis, even among those who had aborted. Therefore, the study focused on clinical manifestations, diagnostic implications and epidemiological characteristics of this outbreak in cattle associated with *L. interrogans* serogroup Pomona. The outbreak occurred in the Northeast of Sicily (Italy), in a protected natural area within the Nebrodi Park. The area is characterized by a multi-host breeding system with the simultaneous presence of several animal species (cattle, sheep, goats, pigs, donkeys, equines) and by the uncontrolled and increased proliferation of wild pigs and wild boars.

The high number of positive animals obtained could be related to the semi-extensive or extensive breeding, promoting contacts with wildlife.

The acute phase of the disease (T0), both in farm A and in farm B, was characterized by antibody reaction to different serogroups (Australis Icterohaemorhagiae serogroup, Sejroe serogroup, Pomona serogroup). These results are consistent with the MAT method, as reported by other authors [49]. This can be explained by the test ability to detect both IgG and IgM immunoglobulins simultaneously. IgM (early antibodies) are present mainly in the first weeks of the disease or the acute phase, and interact with different antigens, some shared by several leptospires, thus showing a reduced specificity towards a single serogroup. Because of the lower specificity of antibodies in the acute phase and cross reactions, the attention in this phase was more focused on leptospiral isolation and molecular detection. Specificity increases in the subsequent convalescence/chronicization phase of the infection (T1 and T2) with the IgG predominance (late antibodies). In this phase, the test is better suited to identify the exact serogroup involved in the outbreak.

Even in farms C and F, subjected to a single sampling, MAT detected the presence of *Leptospira* Pomona with a prevalence of 66% and 29.5%, respectively, confirming the circulation of the Pomona serogroup in four of the six farms during the epidemic outbreak. Furthermore, in farm F a significant circulation of the Tarassovi serogroup occurred with a prevalence of 50%, followed by Sejroe and Grippotyphosa, as a further indicator of the presence of *Leptospira* serovars of swine and other wild animal origin [21]. Farms D and E (with farm consistency of two animals each) showed antibody positivity towards serogroups Ballum and Sejroe, respectively, with low antibody titers. However, they did not show any epidemiological significance due to the small number of animals reared and the low antibody titer.

Although congenital jaundice in aborted fetuses has been included among the clinical signs of leptospiral abortion, the fetuses were not subjected to necropsy because they were in an advanced state of degradation, having been recovered 24 h after the abortion, and after any jaundice was visible.

In Italy, a higher prevalence of serogroup Sejroe serovar Hardjo has been detected in cattle, confirming that cattle represent the main maintenance-host for *Leptospira* belonging to this serogroup [30,34] and, in particular, some strains isolated from urine samples were classified as Hardjobovis. In addition, previous studies showed a relatively high number of positive reactions to serogroups Pomona, Grippotyphosa and Bratislava (serogroup Australis) in cattle. Severe infections in cattle due to Pomona serogroup are uncommon and usually occur in young animals. Nevertheless, in Italy, Pomona resulted in the second most commonly isolated serovar in cattle [24,30]. Although this serogroup has been associated mainly with leptospirosis in pigs, considered its natural carriers [50], other species can also be affected, such as dogs, cattle and sheep [51,52,53,54]. Clinical signs in cattle caused by Pomona are generally different from both Hardjo and Hardjobovis infection and, especially in producing cows, fever and lethargy are milder and usually go unnoticed. At the same time, a transient reduction in milk production and/or agalactia may be detected. In pregnant cows, serogroup Pomona is generally associated with abortion [55].

Many wildlife species have been implicated as reservoirs for the bacteria, including red fox (*Vulpes vulpes*) and wild boar (*Sus scrofa*). Among wildlife, wild boar is an important *Leptospira* reservoir and could represent an appropriate indicator for this zoonotic infectious disease. In Sicily, a study of free-roaming semi-wild black swine demonstrated leptospires by PCR targeting the 16S rRNA gene with prevalence of 40% [56].

True foxes are well recognized as *Leptospira* reservoirs, in particular red foxes (*Vulpes vulpes*), but no isolation was performed among them [57].

Red foxes prey on small rodents, notably *Rattus norvegicus*, which is known to be the main reservoir of L. Icterohaemorhagiae serovar.

Surveys conducted throughout Europe have shown differences in the prevalence of leptospirosis in foxes: 26.3% in central and eastern Poland [58], 31.3% in Croatia [59].

Due to their predatory behavior and their varied diet, mainly composed of small mammals, red foxes could also be considered sentinel animals of environmental contamination with leptospires.

Because these “unconventional” hosts share the environment with cattle, the object of this study, they could have played an important role in leptospirosis spread, and further knowledge of them could give new insights into the epidemiology of this infection.

## 5. Conclusions

This study provides the first description of a *Leptospira* outbreak in cattle due to Pomona serovar in a protected natural geographical area of Northeastern Sicily, characterized by a multi-host environment with the presence of different animal species, domestic and wild, sharing pastures and food and water sources. In this ecosystem, where several domestic and wild mammals are natural reservoirs of pathogenic leptospires, and where appropriate management of wild pigs and boars is lacking, further investigations are required to confirm the role of domestic and wild species in the transmission, diffusion and persistence of the Pomona serovar among cattle farms.

## Figures and Tables

**Table 1:** Commercially available ELISA test kits used for detecting antibodies against *N. caninum*, *C. burnetii*, IBR and BVD, to assess the cause of abortion in the cattle herds. * The sensitivity (Se) and specificity (Sp) of the diagnostic kits were provided by the manufacturer of the kits.

Infectious Agent	ELISA Test Kit	Manufacturer	Antigens	Se *	Sp *
*Neospora caninum*	ID Screen^®^ *Neospora caninum* Indirect Multi-species	ID.vet Innovative Diagnostics, Grabels, France	Purified extract of *Neospora caninum*	100% (CI95%: 98.8–100%)	100% (CI95%: 99.41–100%)
*Coxiella burnetii*	ID Screen^®^ Q fever indirect Multi-species	ID.vet Innovative Diagnostics, Grabels, France	phase I and phase II antigens *Coxiella burnetii*	100% (CI95%: 89.28–100%)	100% (CI95%: 97.75–100%)
Infectious Bovine Rhinotracheitis Virus	Infectious Bovine Rhinotracheitis Virus (BHV1) gB Antibody Test Kit	IDEXX Laboratories, Inc.	Bovine herpesvirus-1 specific Glycoprotein-B (gB)	99.6% (CI95% 98.0–99.9%)	100.0% (CI95% 99.5–100.0%)
	Infectious Bovine Rhinotracheitis Virus (BHV-1) gE Antibody Test Kit		Bovine herpesvirus-1 specific Glycoprotein-E (gE)		
Bovine Viral Diarrhoea	Bovine Viral Diarrhoea Virus (BVDV) Antigen Test Kit/Serum Plus	IDEXX Laboratories, Inc.	Bovine Viral Diarrhoea Virus E-antigen	95.9% (CI95% 92.3–97.9%)	100% (CI95% 97.7–100%)

**Table 2:** Nucleotide sequences of primers and hydrolysis probe used for the amplification of a *lipL32* gene fragment from pathogenic *Leptospira* species.

Oligonucleotide	Target	Sequence 5′–3′	Refererence
LipL32-45F	*lipL32*	5′-AAGCATTACCGCTTGTGGTG-3′	[37]
LipL32-286R	*lipL32*	5′-GAACTCCCATTTCAGCGATT-3′	
LipL32-189P	*lipL32*	FAM-5′-AAAGCCAGGACAAGCGCCG-3′-BHQ1	

**Table 3:** MAT results in cows from Farm A at T0, T1 and T2 (Cut-off ≥ 100). Abbreviations: M, male; Neg, negative; Antib, subjected to antibiotic treatment. The arrows ↑↓ indicate an increase (↑) or a reduction (↓) of the antibody titer respect to the previous sampling, the (=) is used in the case of no variation, and the (I) sign indicates the first detection at T1 of Pomona serogroup for the serologically negative animals at T0.

ID	T0		ANTIB	T1			T2		
	Serogroup	Titer		Serogroup	Titer	↑↓	Serogroup	Titer	↑↓
1A	Australis Icterohem. Pomona	1:400 1:400 1:200	Yes	Pomona	1:6400	↑	Pomona	1:1600	↓
2A	Icterohem. Pomona	1:100 1:100	Yes	Pomona	1:3200	↑	Pomona	1:1600	↓
3A	Pomona	1:100	Yes	Pomona	1:800	↑	Pomona	1:400	↓
4A	Pomona	1:100	Yes	Pomona	1:1600	↑	Pomona	1:1600	=
5A	Australis Icterohem. Pomona	1:100 1:400 1:400	Yes	Pomona	1:800	↑	Pomona	1:3200	↑
6A	Icterohem. Pomona	1:100 1:200	Yes	Pomona	1:400	↑	Neg		↓
7A	Icterohem.	1:200	Yes	Pomona	1:100	I	Pomona	1:6400	↑
8A	Tarassovi	1:200	Yes	Pomona	1:200	I	Pomona	1:200	=
9A	Australis Icterohem. Pomona	1:200 1:200 1:400	Yes	Pomona	1:6400	↑	Pomona	1:3200	↓
10A	Pomona	1:200	Yes	Pomona	1:100	↓	Neg		↓
11A	Icterohem.	1:400	Yes	Neg			Pomona	1:800	↑
12A	Neg			Pomona	1:6400	I	Pomona	1:400	↓
13A	Neg			Pomona	1:1600	I	Pomona	1:800	↓
14A	Neg			Pomona	1:200	I	Pomona	1:800	↑
15A	Neg			Pomona	1:100	I	Pomona	1:1600	↑
16A	Neg			Pomona	1:6400	I	Pomona	1:3200	↓
17A	Neg			Pomona	1:800	I	Pomona	1:200	↓
18A	Neg			Pomona	1:200	I	Pomona	1:400	↑
19A	Neg			Neg		=	Neg		=
20A	Neg			Neg		=	Neg		=
21A	Neg			Dead					
22A	Neg			Pomona	1:200	I	Neg		↓
23A	Neg			Pomona	1:100	I	Pomona	1:100	=
24 M	Neg.			Neg.		=	Neg		=
25A	Neg			Pomona	1:200	I	Neg		↓
26A	Neg			Pomona	1:800	I	Pomona	1:200	↓
27A	Neg			Pomona	1:800	I	Pomona	1:800	=
28A	Neg			Pomona	1:100	I	Neg		↓
29A	Neg			Pomona	1:800	I	Pomona	1:800	=
30A	Neg			Pomona	1:6400	I	Pomona	1:800	↓
31A	Neg			Neg		=	Neg		=
32A	Neg			Neg		=	Pomona	1:200	↑
33A	Neg			Neg		=	Neg		=

**Table 4:** MAT results in cows from Farm B at T0 and T1 (Cut-off ≥ 100). Abbreviations: Neg, negative. The arrows ↑↓ indicate an increase (↑) or a reduction (↓) of the antibody titer respect to the previous sampling, and the (I) sign indicates the first detection of Pomona serogroup.

ID	T0		T1		↑↓
	Serogroup	Titer	Serogroup	Titer	↑
1B	Australis Icterohaem Sejroe Pomona	1:400 1:800 1:100 1:3200	Pomona	1:6400	
2B	Pomona	1:1600	Pomona	1:800	↓
3B	Pomona Icterohaem Sejroe	1:400 1:400 1:100	Pomona	1:800	↑
4B	Pomona	1:800	Pomona	1:800	↓
5B	Sejroe	1:100	Neg		↓
6B	Neg		Pomona	1:200	I
7B	Neg		Pomona	1:200	I

**Table 5:** Cattle from farms C, D, E and F positive at MAT at T0.

Farm	ID	Serogroup	Titer
C	1C	Ballum	1:100
C	2C	Pomona	1:200
C	3C	Pomona	1:800
D	1D	Ballum	1:100
E	1E	Sejroe	1:200
F	1F	Grippotyp/Sejroe/Tarassovi	1:400/1:200/1:100
F	2F	Grippotyp/Sejroe/Tarassovi	1:100/1:200/1:400
F	3F	Grippotyp/Sejroe/Tarassovi/Pomona	1:400/1:100/1:100/1:400
F	4F	Grippotyp/Sejroe	1:400/1:800
F	5F	Grippotyp/Tarassovi	1:200/1:400
F	6F	Grippotyp/Sejroe	1:400/1:400
F	7F	Grippotyp/Sejroe/Tarassovi	1:100/1:200/1:100
F	8F	Grippotyp/Sejroe/Tarassovi	1:1600/1:1600/1:800
F	9F	Grippotyp/Sejroe	1:400/1:400
F	10F	Grippotyp	1:800
F	11F	Grippotyp/Tarassovi/Pomona	1:100/1:200/1:800
F	12F	Grippotyp/Tarassovi	1:800/1:100
F	13F	Pomona/Sejroe/Tarassovi	1:400/1:200/1:400
F	14F	Pomona/Sejroe	1:100/1:400
F	15F, 23F	Pomona	1:400
F	16F	Tarassovi/Pomona	1:400/1:200
F	17F, 22F	Pomona	1:800
F	18F	Pomona/Sejroe	1:400/1:200
F	19F	Pomona	1:1600
F	20F	Pomona/Sejroe	1:200/1:100
F	21F	Tarassovi/Pomona	1:200/1:3200
F	24F, 26F	Sejroe	1:100
F	25F, 30F, 32F, 33F	Sejroe	1:200
F	27F	Tarassovi/Sejroe	1:400/1:400
F	28F, 31F	Sejroe	1:400
F	29F	Tarassovi/Sejroe	1:200/1:400
F	34F, 37F, 40F	Tarassovi	1:400
F	35F, 38F, 41F, 42F, 43F, 44F	Tarassovi	1:200
F	36F, 39F	Tarassovi	1:100

**Table 6:** Results of Real Time-PCR, MLST and isolation from urine samples collected in the acute phase (T0). Abbreviations: Pos, positive; Neg, negative; /, not investigated, Cs, contaminated sample. No urine samples were collected in the farm F.

Farm	ID	Real Time-PCR	MLST	Isolation
A	1A	Pos	Pos	Pos
A	2A	Pos	/	Neg
A	3A	Pos	Pos	Pos
A	4A	Pos	/	Neg
A	5A	Pos	Pos	Neg
A	6A	Pos	Pos	Neg
A	7A	Pos	/	Neg
A	8A	Pos	Pos	Neg
A	9A	Pos	Pos	Neg
A	10A	Pos	Pos	Neg
A	11A	Pos	/	Neg
B	1B	Neg	/	Pos
B	2B	Neg	/	Neg
B	3B	Neg	/	Neg
B	4B	Neg	/	Neg
B	5B	Neg	/	Neg
C	1C	Neg	/	Pos
C	2C	/	/	Neg
C	3C	Neg	/	Neg
D	1D	Neg	/	Cs
E	1E	Neg	/	Cs

## Data Availability

The data that support the findings of this study are available from the corresponding author upon reasonable request.
